# Clear Cell Renal Cell Carcinoma Involving Intrarenal Adrenal Tissue: A Rare Case With Staging Implications

**DOI:** 10.7759/cureus.101640

**Published:** 2026-01-15

**Authors:** Aarohi Shah, Benjamin Spilseth, Paari Murugan

**Affiliations:** 1 Department of Laboratory Medicine and Pathology, University of Minnesota, Minneapolis, USA; 2 Department of Radiology, University of Minnesota, Minneapolis, USA

**Keywords:** adrenal-renal fusion, clear cell renal cell carcinoma, ectopic adrenal tissue, intrarenal adrenal rest, tumor staging

## Abstract

The presence of intrarenal adrenal tissue is a rare finding that complicates the staging of renal cell carcinoma, particularly in distinguishing between adrenal-renal fusion and ectopic adrenal remnants. These conditions, though rare, have unique implications for tumor staging and management. We present a case of a 47-year-old woman with a lesion of the right kidney incidentally discovered during imaging for ulcerative colitis. Histopathology revealed clear cell renal cell carcinoma infiltrating adrenal tissue confined to the kidney capsule, with no lymphovascular or perirenal fat invasion. Radiologic correlation indicated ectopic rather than fused adrenal tissue. Failure to correctly classify the presence of adrenal involvement could result in incorrect staging of the carcinoma, highlighting the importance of integrating histopathologic and radiologic findings in accurate diagnosis and management.

## Introduction

Ectopic adrenal tissue has been described in many anatomic locations, including the kidney, liver, lung, brain, testis, and mesoappendix [1]. The ectopic adrenal tissue, or adrenal rest (AR), may undergo significant hyperplasia and subsequently give rise to adenomas or carcinomas [1,2]. Intrarenal adrenal tissue occurs in approximately 1% of the adult population, typically discovered incidentally during nephrectomy or autopsy, with most cases having minimal clinical significance unless tumors arise [2]. While adrenocortical neoplasms arising within ectopic adrenal tissue have been reported, to our knowledge, this text represents the first documented case of clear cell renal cell carcinoma (ccRCC) infiltrating intrarenal ectopic adrenal tissue, presenting unique diagnostic and staging challenges. We explore a case of ccRCC infiltrating intrarenal AR and investigate the nuances of staging this tumor along with the utility of both histopathology and radiology in diagnosis. Understanding the embryologic origin and anatomical relationship between the kidney and adrenal gland is crucial to understanding how ectopic adrenal tissue may become embedded in the kidney and give rise to pathologic conditions.

The embryologic development of the kidneys and adrenal glands occurs separately. The kidneys originate from the intermediate mesoderm, progressing from pronephros to mesonephros to metanephros before definitively becoming the kidney [3]. The adrenal cortex arises from the coelomic mesoderm of the urogenital ridge, while the adrenal medulla arises from neural crest cells [4]. The kidneys are enveloped by a fibrous capsule that is surrounded by perirenal fat and further enclosed by Gerota fascia, which provides anchoring support for the kidneys [3]. Superiorly, atop each kidney and separated by Gerota fascia is an adrenal gland surrounded by its own capsule [3,4]. Despite their close anatomical proximity, the kidney and adrenal gland maintain individual functions bilaterally. Most cases of intrarenal AR are discovered incidentally through autopsy or nephrectomy and have no clinical significance, unless the tissue gives rise to tumors [1,2]. Intrarenal AR is typically located at the superior poles of the kidney, with well-maintained adrenal gland architecture [2].

## Case presentation

**Figure 1:**
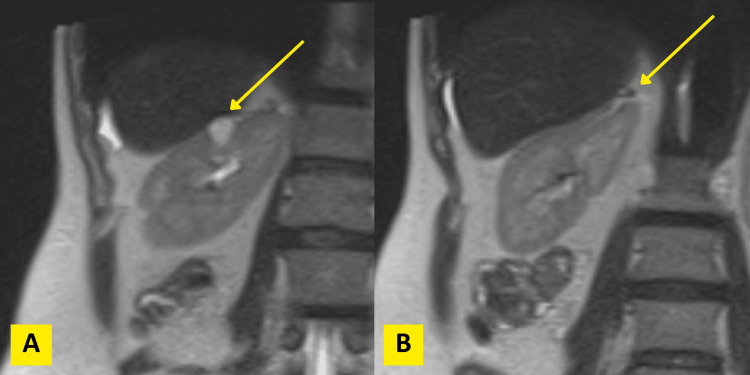
MR enterography demonstrating a small, atrophic right adrenal gland abutting the renal capsule but separate from the renal mass (A, B). MR: magnetic resonance

**Figure 2:**
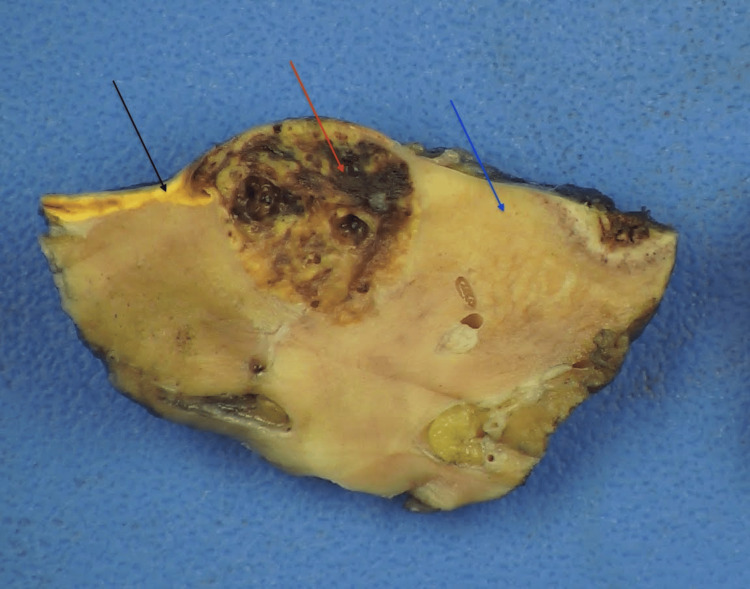
Macroscopic findings showing adrenal cortical tissue (black arrow), with involvement by ccRCC (red arrow), and normal renal parenchyma (blue arrow). ccRCC: clear cell renal cell carcinoma

**Figure 3:**
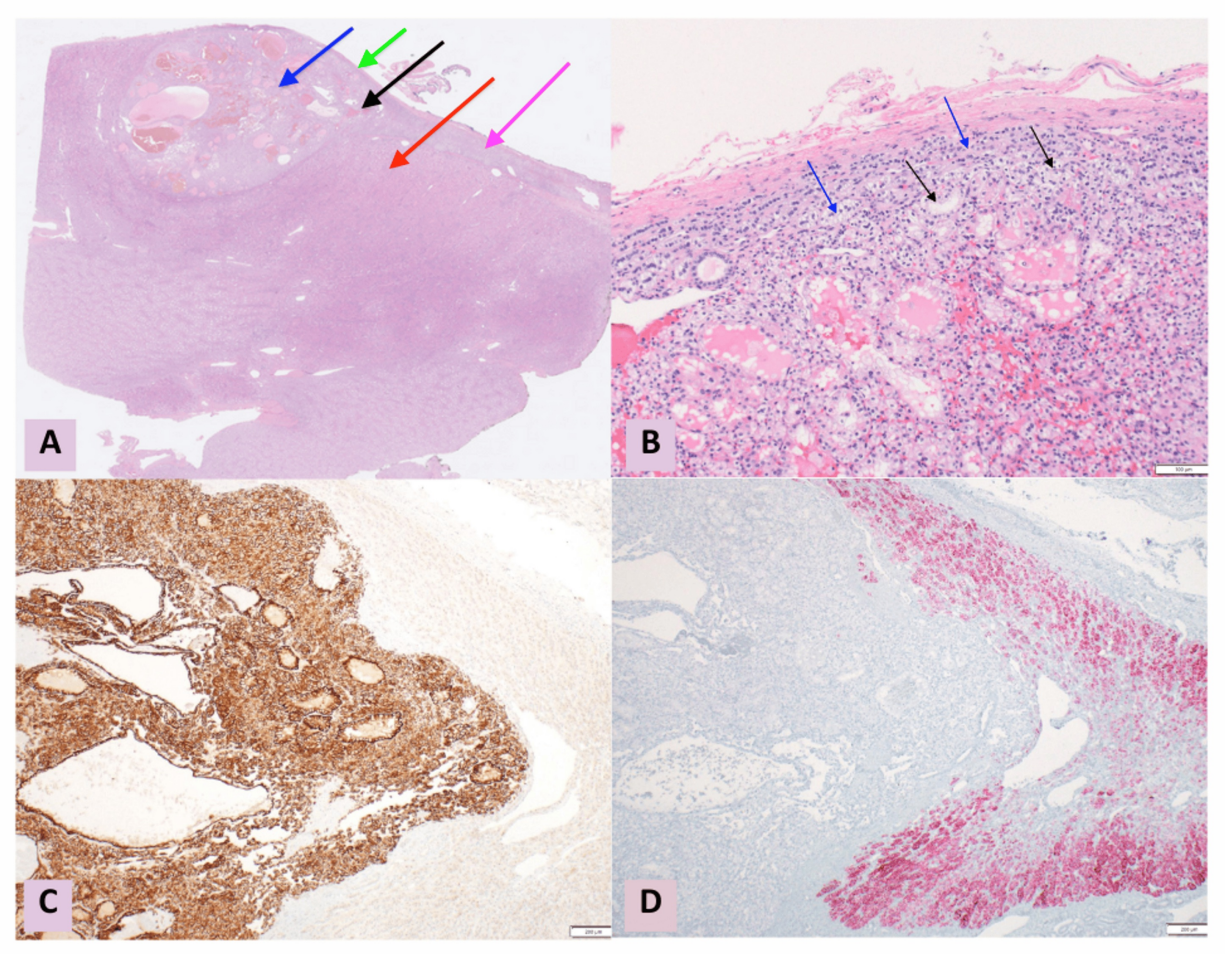
Microscopic findings. (A) Renal cortical ccRCC (blue and black arrows) involving ectopic adrenal cortical tissue (pink arrow) with adjacent renal capsule (green arrow) and normal renal parenchyma (red arrow) (magnification 12.5x). (B) Magnified view of adrenal cortical tissue (blue arrow) and infiltrating ccRCC (black arrow) (magnification 100x). (C) Carbonic anhydrase IX stain shows diffuse reactivity in the ccRCC while the adjacent adrenal cortical tissue is negative (magnification 40x). (D) Melan-A stain highlights the adrenal cortical tissue (magnification 40x). ccRCC: clear cell renal cell carcinoma

A 47-year-old female patient with a history of ulcerative colitis presented with a 1.5 cm upper pole lesion of the right kidney, incidentally discovered during imaging for ulcerative colitis evaluation. On magnetic resonance (MR) enterography (Figure 1), the right adrenal gland appeared small, atrophic, and irregularly shaped, with its upper pole abutting the renal capsule but distinct from the mass. Functional activity (cortisol production) from the ectopic adrenal cortical tissue likely resulted in native adrenal gland atrophy due to feedback suppression. The patient denied hematuria and flank pain and had no known family history of kidney cancer. She underwent robotic partial nephrectomy (Figure 2), yielding clear tumor margins. Histopathology revealed ccRCC confined to the kidney without lymphovascular invasion or perirenal/renal sinus fat invasion. Interestingly, intrarenal ectopic adrenal tissue was noted underneath the renal capsule. This extremely rare phenomenon was complicated by the renal carcinoma abutting and infiltrating the ectopic adrenal cortical tissue as noted on the histomorphologic examination, aided by differentiating immunohistochemical markers (Figure 3).

## Discussion

Although current literature does not clearly differentiate these two entities, it is important to note that adrenal-renal fusion is a discrete diagnosis from intrarenal AR due to their differing embryologic origins [2,5]. It is hypothesized that the congenital form of adrenal-renal fusion is a result of a retroperitoneal mesenchymal defect that fails to stimulate the formation of the adrenal capsule [6,7]. This fusion refers to a condition where the adrenal tissue found within the renal capsule is functioning as the sole adrenal gland. The fused adrenal gland is effectively replacing the conventional adrenal gland function, meaning there would be an absence of the ipsilateral adrenal gland to the kidney with the adrenal-renal fusion. Conversely, intrarenal AR is likely to develop from fragments of primitive adrenal glands that shed during development [2]. This ectopic tissue is located within the kidney’s capsule but does not replace the function of the existing adrenal glands. Unlike adrenal-renal fusion, in cases of intrarenal ectopic adrenal tissue, both bilateral adrenal glands would be present without anatomic variation and would be fully functioning. This case presents radiologic findings with two normal bilateral adrenal glands, while histology reveals intrarenal adrenal tissue enclosed in the kidney’s capsule. This correlation indicates that the adrenal tissue discovered is ectopic, rather than an adrenal-renal fusion. Because both embryologic entities are confined to the kidney capsule, our diagnosis will not change the tumor staging with respect to the relationship of renal carcinomas to the adrenal gland.

This case is unique in that it is the first description of ccRCC involving an intrarenal AR. Ye et al. reported nine cases of intrarenal adrenal tissue and renal-adrenal fusion, all composed solely of adrenal cortical tissue without medullary components and primarily located at the superior pole [2]. In their series, intrarenal adrenal tissue occasionally mimicked low-grade ccRCC when normal adrenal architecture was lost or when clear cells predominated, highlighting the diagnostic challenge. Two cases posed particular diagnostic difficulties: one where intrarenal adrenal tissue deep in the renal parenchyma mimicked ccRCC due to its prominent vascular network, and another where an adrenocortical adenoma arising in renal-adrenal fusion raised concern for adrenocortical carcinoma invading the kidney [2]. While the author described diagnostic pitfalls in distinguishing adrenal tissue from renal cell carcinoma (RCC), our case presents the novel scenario of actual ccRCC infiltrating ectopic adrenal tissue. Histopathologic differentiation relies on immunohistochemistry: RCC typically expresses carbonic anhydrase IX and is negative for inhibin and Melan-A, while adrenal cortical tissue demonstrates the opposite immunoprofile [1,2]. Our case highlights the importance of integrating immunohistochemical findings with radiologic confirmation of bilateral normal adrenal glands, as misinterpretation may lead to inappropriate staging.

ccRCC in the setting of intrarenal AR, as seen in this patient, must be staged appropriately. In the most recent “Protocol for the Examination of Resection Specimens From Patients With Renal Cell Carcinoma” published by the College of American Pathologists, conventions explain the importance of determining the extent of renal carcinoma invasion for appropriate staging and prognostication purposes [8]. A tumor that is less than or equal to 7 cm in dimension and also limited to the renal parenchyma is labeled as primary tumor (pT) 1, either “a” or “b” depending on the size. Invasion of the tumor into renal sinus tissue, renal vein, or perirenal fat is classified as pT3, while Gerota fascia as well as ipsilateral adrenal gland involvement is considered pT4. In particular, emphasis is placed on contiguous versus non-contiguous adrenal gland involvement, with the former necessitating pT4 tumor stage while the latter is considered pM1. The assignment of pT4 stage for contiguous adrenal gland involvement is based on the fact that the adrenal is separated from and enveloped by the Gerota fascia, and direct renal tumor infiltration into the adrenal would imply tumor growth through the overlying Gerota fascia [8].

In our case, given that the tumor remains confined within the renal capsule, which includes both adrenal and renal tissue, it is crucial to accurately stage this carcinoma. By classifying this tumor as pT1a, we underscore the tumor’s limited size (≤4 cm) and containment within the kidney parenchyma, emphasizing its relatively lower stage compared to tumors that invade beyond the renal capsule. The classification not only reflects the tumor’s current state but also has significant implications for prognosis and treatment strategies. Accurate staging from a pathologist is essential to guide clinical decisions. Additionally, in situations where radiologic information regarding the adrenal gland is not available, such tumors might be incorrectly staged, potentially leading to a pT4 misclassification.

## Conclusions

When faced with unusual presentations, by integrating pathology, radiology, and knowledge of variations in adrenal gland anatomy/embryology, we ensure the patient receives relevant care tailored to the behavior of their carcinoma. Although these conclusions are drawn from a single case, this report highlights critical diagnostic and staging considerations that may be encountered in similar rare presentations, emphasizing the importance of multidisciplinary correlation to guide optimal patient management. Failure to correctly classify the presence of adrenal involvement could result in incorrect staging of the carcinoma, highlighting the importance of integrating histopathologic and radiologic findings in accurate diagnosis and management.
